# Profiling of Intestinal Microbiota in Patients Infected with Respiratory Influenza A and B Viruses

**DOI:** 10.3390/pathogens10060761

**Published:** 2021-06-17

**Authors:** Hebah A. Al Khatib, Shilu Mathew, Maria K. Smatti, Nahla O. Eltai, Sameer A. Pathan, Asmaa A. Al Thani, Peter V. Coyle, Muna A. Al Maslamani, Hadi M. Yassine

**Affiliations:** 1 Biomedical Research Center, Qatar University, Doha 2713, Qatar; h.alkhatib@qu.edu.qa (H.A.A.K.); shilu.mathew@qu.edu.qa (S.M.); msmatti@qu.edu.qa (M.K.S.); nahla.eltai@qu.edu.qa (N.O.E.); aaja@qu.edu.qa (A.A.A.T.); 2 Emergency Medicine, Hamad Medical Corporation, Doha 3050, Qatar; SPATHAN@hamad.qa; 3 Department of Biomedical Sciences, College of Health Sciences, Qatar University, Doha 2713, Qatar; 4 Virology Laboratory, Hamad Medical Corporation, Doha 3050, Qatar; PCoyle@hamad.qa; 5 Communicable Diseases Center, Hamad Medical Corporation, Doha 3050, Qatar; MALMASLAMANI@hamad.qa

**Keywords:** influenza, intestine microbiome, diversity, virus shedding

## Abstract

Little is known about the association between respiratory viral infections and their impact on intestinal microbiota. Here, we compared the effect of influenza types, A and B, and influenza shedding in patients’ stools on the gut microbiota diversity and composition. Deep sequencing analysis was performed for the V4 region of the 16S rRNA gene. Fecal samples were collected from 38 adults with active respiratory influenza infection and 11 age-matched healthy controls. Influenza infection resulted in variations in intestinal bacterial community composition rather than in overall diversity. Overall, infected patients experienced an increased abundance of *Bacteroidetes* and a corresponding decrease in *Firmicutes*. Differential abundance testing illustrated that differences in gut microbiota composition were influenza type-dependent, identifying ten differentially abundant operational taxonomic units (OTUs) between influenza A- and influenza B-infected patients. Notably, virus shedding in fecal samples of some patients had significantly reduced gut bacterial diversity (*p* = 0.023). Further taxonomic analysis revealed that the abundance of *Bacteroides fragilis* was significantly higher among shedders compared to non-shedders (*p* = 0.037). These results provide fundamental evidence of the direct effect of influenza infection on gut microbiota diversity, as reported in patients shedding the virus.

## 1. Introduction

Susceptibility to infections and disease severity is controlled by a complex relationship between pathogen, host, and environment. Accordingly, studies evaluating the contribution of these three factors in infection development and severity are being conducted worldwide. Recently, researchers started investigating the role of the host metagenome in pathogen-virus interaction. Humans host a considerable number of symbiotic bacteria on most surfaces, such as the intestinal and respiratory tracts [1,2]. The human gastrointestinal tract, particularly, harbors the most complex and dynamic bacterial populations in the human body [3]. The intestinal microbiota plays an essential role in maintaining the intestinal environment by promoting mucosal development, helping in nutrient metabolism, and protecting against pathogenic bacteria [4,5]. Importantly, the role of intestinal microbiota also extends to influence systems beyond the gastrointestinal tract by modulating the immune response [6,7,8,9,10,11].

Consequently, alteration of intestinal commensal bacteria balance (also known as dysbiosis) could result in significant consequences for human health. Although it is widely accepted that diet is the major contributor in shaping gut microbiota [12,13], the type and duration of medications, especially antibiotics, can significantly affect gut microbiota composition [14]. Several viral and bacterial infections were also identified as a disturbing factor of microbiota balance [15,16,17,18]. Moreover, intestinal dysbiosis has also been linked to increased susceptibility to respiratory infections [19,20]. The depletion of the gut microbiota in mice was linked to reduced antibody-secreting cells and virus-specific T cells, which resulted in reduced responses to influenza virus infection and vaccination [4,8,21].

Respiratory viral infections are highly prevalent, yet only a few studies have explored the impact of respiratory infections on the intestinal microbiota [18,22,23]. Respiratory viruses such as influenza [22], respiratory syncytial virus [23], SARS coronavirus [24], adenovirus [25], and bocavirus [26] have been detected in fecal samples from patients with upper respiratory tract infections. Moreover, gastrointestinal (GI) symptoms have been reported in patients with pandemic and seasonal influenza, especially among patients infected with the avian influenza H5N1 virus [27,28,29,30]. For instance, an early epidemiological study of the pandemic influenza H1N1 virus in 2009 reported GI symptoms in 25% of patients and in the stools of hospitalized and outpatients with respiratory manifestations [31,32,33]. Yet, the mechanism that mediates GI pathogenesis in influenza-infected patients is not fully understood. Multiple explanations were proposed, such as the GI symptoms could appear as a side effect of antibiotic treatment, intestinal microbiota imbalance, or co-infection with other intestinal pathogens [34]. More importantly, the bidirectional effect of the “gut-lung axis” is still widely debatable, and the impact of lung infections on gut microbiota is the target of many research studies in the last few years [35]. In this context, we conducted this study to investigate the effect of influenza types (A and B) on gut microbiota regardless of GI symptoms, and to understand the possible association of microbiota profiles with the severity of respiratory symptoms.

## 2. Results

### 2.1. The Composition of the Intestinal Microbiota Is Affected during Influenza Infection

**Figure 1:**
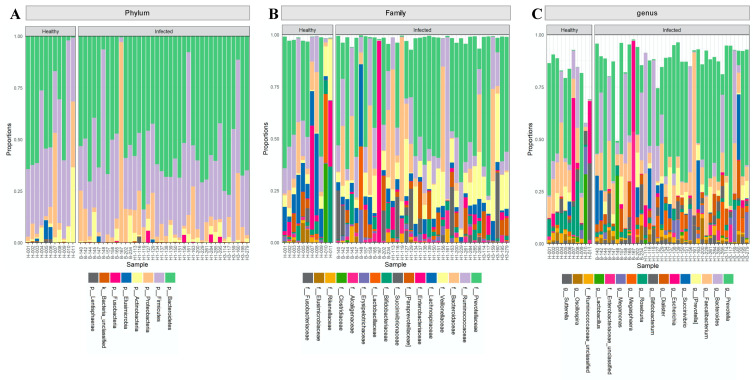
Overall relative abundance of OTUs identified in all samples included in the study. A total of 8 phyla, top 16 families and the top 16 genera are presented. (**A**) Phylum level (**B**) Family level and (**C**) Genus’s level.

**Figure 2:**
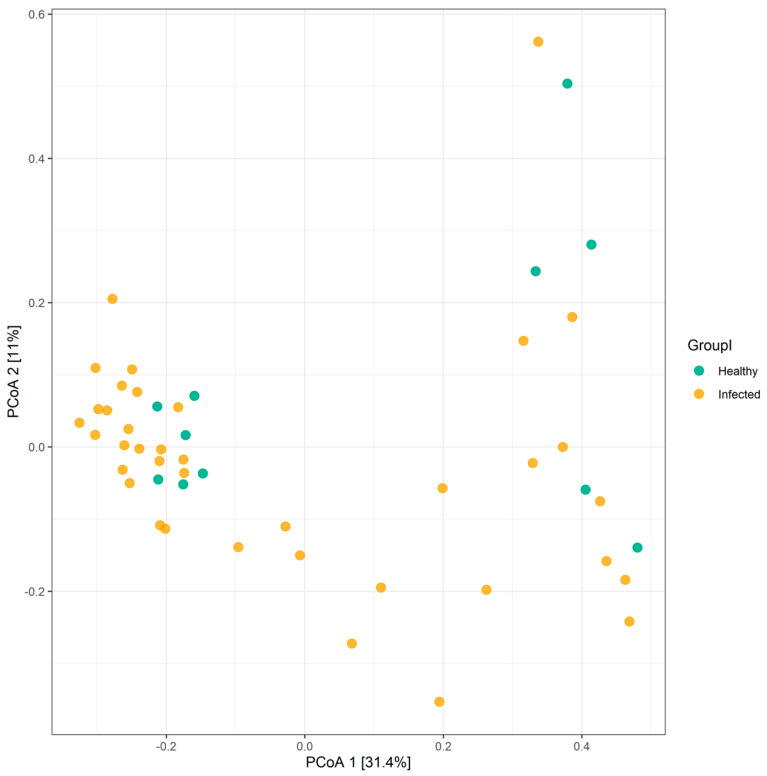
Beta diversity plot of microbiome composition similarity among all samples included in the study. The Bray–Curtis index was used to generate a PCoA ordination and visualize dissimilarities between healthy controls (green) and influenza-infected patients (orange). Each dot denotes microbiota profile of a single individual in a low-dimensional space in which samples cluster together based on their microbiome profiles.

**Figure 3:**
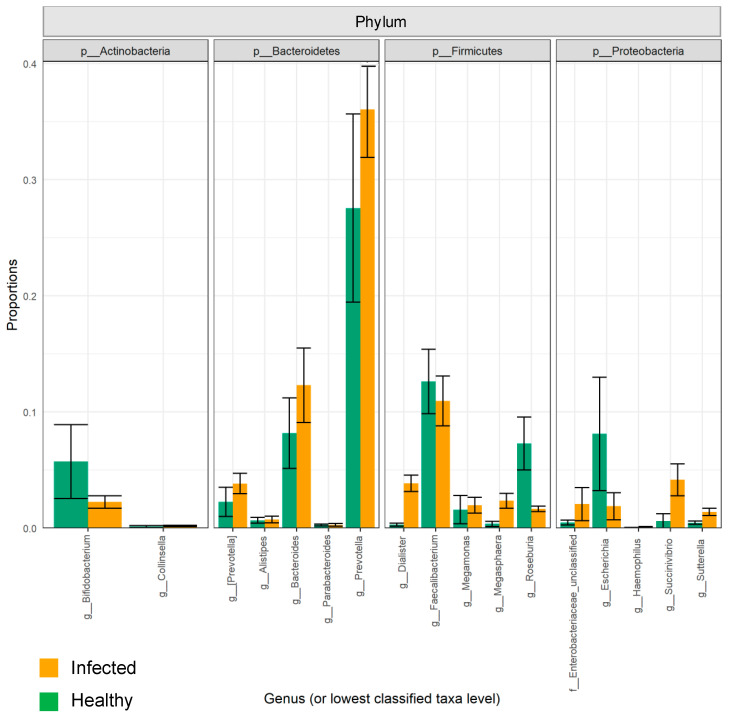
The relative abundances of the five most abundant genus-level taxa within the four most abundant Phyla found in influenza-infected individuals (orange) and healthy controls (green). The plot illustrates the mean and standard error of each of genus-level taxa. The X-axis denotes the Genus (or lowest classified taxa level) and the Y-axis denotes proportions.

**Figure 4:**
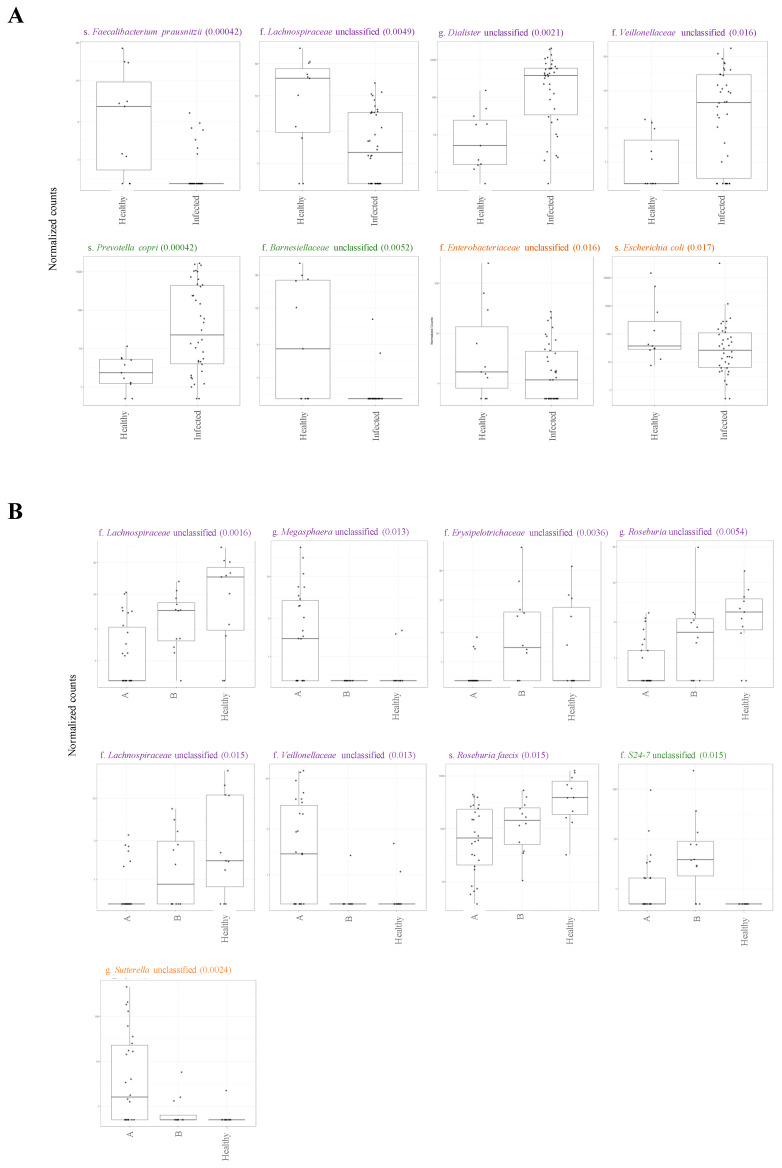
Differentially abundant OTUs identified in (**A**) influenza-infected patients and healthy controls (**B**) influenza A, influenza B and healthy controls. Differentially abundant OTUs were identified amongst samples of each set of groups using a DESeq2 package. The most featured differentially abundant OTUs between patients and healthy controls (panel **A**) and between influenza A- and influenza B-infected patients (panel **B**). OTUs are colored based on phyla as follows: purple for Firmicutes, green for Bacteroidetes and orange for Proteobacteria. The number between brackets represents the adjusted *p* value (Padj value threshold = 0.05).

Overall, the influenza A virus was detected in 26 patients, of which 20 were H1N1 positive. Influenza B was detected in nasal samples of 12 participants (Table 1). None of the patients included in this study had a co-infection. First, we investigated the impact of influenza infection on the abundance, richness, and composition of the gut microbiota. Generally, influenza infection showed no significant effect on the total number of observed OTUs in infected individuals compared to healthy individuals. A total of 8 phyla and 73 genera were reported in influenza-infected individuals compared to 7 phyla and 70 genera in controls (Figure 1). Moreover, no significant differences (*p* = 0.348) were reported when comparing alpha diversity of infected individuals (mean = 3.068; SD = 0.771) and healthy controls (mean = 3.307; SD = 0.592) as determined by ANOVA test. However, there was a significant difference in beta diversity (*p* < 0.04) amongst samples of the two groups (Figure 2). Therefore, we decided to compare gut microbiota composition between the groups by dissecting them at the phyla level. The dominant phyla in all individuals, healthy and infected, were *Bacteroidetes* and *Firmicutes* (~85–95% combined total relative abundance), followed by *Actinobacteria* and *Proteobacteria*, yet to a much lesser extent (~1–25%) (Figure 3). Other phyla were also sporadically detected in some individuals, such as *Elusimicrobia*, *Fusobacteria,* and *Lentisphaerae,* but these individually never exceeded ~5% relative abundance. Overall, we identified 15 OTUs showing significant differences in their relative abundance in the gut bacterial community of healthy compared infected individuals (Supplementary Table S1). A comparison of the dominant phyla in influenza-infected individuals revealed an increase in the relative abundance of *Bacteroidetes* phylum. This increase was driven by a significant increase in *Prevotella copri* in infected patients (*p* < 0.001; Figure 4) compared to healthy controls. The decrease in *Firmicutes*, on the other hand, was associated with a significant decrease in the relative abundance of the three unclassified *Lachnospiraceae* families (*p* < 0.05) as well as the *Ruminococcaceae* family represented by the *Faecalibacterium_prausnitzii* (*p* < 0.001). Despite the overall decrease in *Firmicutes,* a significant increase (*p* < 0.05) in three *Dialister* genera of the *Firmicutes* phylum was also reported in infected individuals (Figure 4).

### 2.2. Variations in Gut Microbiota Composition Are Directly Related to Influenza Type

Here, we subcategorized influenza-positive individuals based on their types of influenza A- and influenza B-infected individuals. We investigated whether the above-reported variations in gut microbiota composition are associated with one influenza type rather than the other. However, the analysis of alpha and beta diversity of patients infected with either type revealed no significant differences between the two groups. Conversely, the influenza virus types significantly affected the differential abundance of certain families and genera (Supplementary Table S2). In both groups, the overall reduction in Firmicutes phylum was associated with a significant decrease in three unclassified families of Lachnospiraceae (*p* < 0.05), one unclassified genus of Roseburia (*p* *=* 0.015), and Roseburia faecis species (*p =* 0.015) as compared to healthy controls reduction was more noticeable among influenza A-infected individuals (Figure 4B). There was an increase in the relative abundance of specific families belonging to the Firmicutes phylum in both groups; however, it was driven by the expansion/reduction in different families. During influenza B virus infection, for example, there was a significant increase in the relative abundance of the Erysipelotrichaceae family (*p* = 0.004) compared to influenza A-infected individuals and healthy controls. Influenza B-infected individuals have also demonstrated a corresponding decrease in an unclassified Megasphaera genus (*p* = 0.013) as well as in an unclassified Veillonellaceae family (*p =* 0.013), both belonging to Firmicutes phylum. In contrast, the latter two OTUs were found to be significantly abundant among individuals infected with influenza A compared to both influenza B-infected individuals and healthy controls (Figure 4B). Moreover, differences in the differential abundance of specific families and genera belonging to other phyla were also reported between the two groups. While the above-reported Bacteroidetes expansion in infected individuals was primarily due to the increase in the relative abundance of Prevotella copri, influenza B-infected individuals, specifically, showed a significant additional increase in the S24_7 family (*p =* 0.015; Figure 4B) that belong to the same phylum. Families and genera of Proteobacteria were also detected at variable abundances between the two groups. While influenza A-infected individuals had a significant increase in the unclassified Proteobacteria phylum, Sutterella genus (*p =* 0.002; Figure 4B), influenza B-infected individuals had a relatively increased abundance of Enterobacteriaceae-unclassified and Escherichia coli. Therefore, although influenza type did not have an impact on the overall microbiota diversity, it had a significant effect on microbiota composition as reported by differential abundance testing.

### 2.3. Shedding of H1N1 Influenza Virus in Stools of Infected Individuals Alters Gut Microbiota Diversity

**Figure 5:**
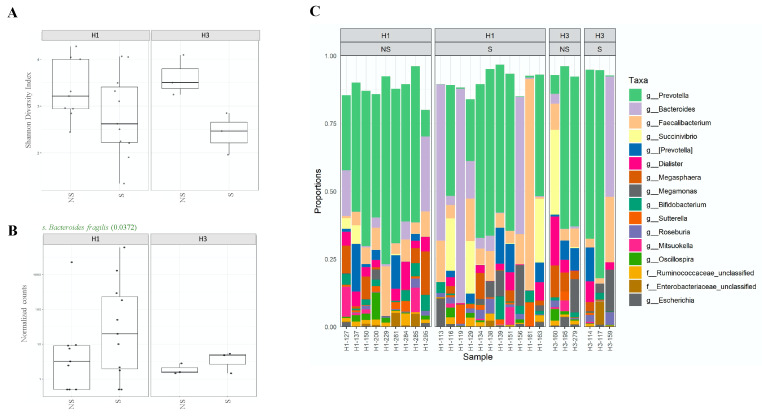
(**A**) Plots presenting alpha diversity (Shannon index) of intestinal microbiota of shedders (S) and non-shedders (NS) among H1N1- and H3N2-infected patients. (**B**) Relative abundances of the five most abundant genus-level taxa within the four most abundant genus-level taxa within the four most abundant Phyla among H1N1- and H3N2-infected patients. (**C**) Differentially abundant OTUs identified in shedders and non-shedders of H1N1- and H3N2-infected patients.

**Figure 6:**
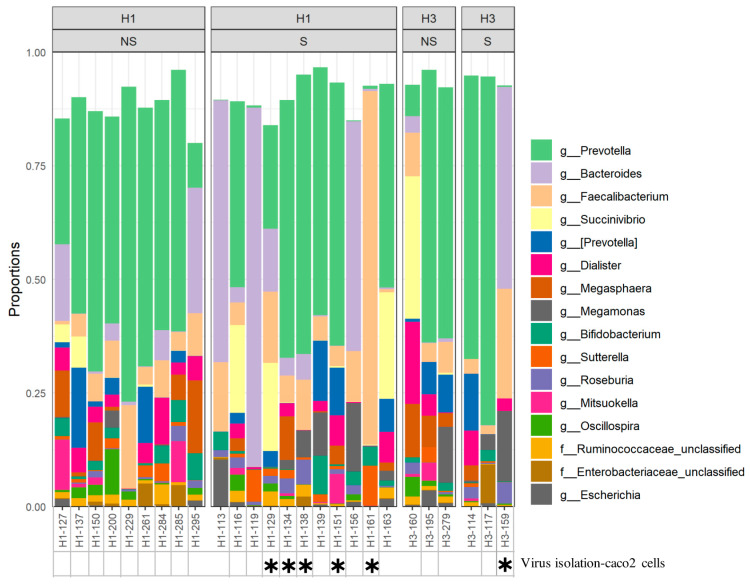
Microbial profiles of shedders and non-shedders among H1N1-and H3N2-infected patients. OTUs are classified at the genus level. Black stars—below the figure- denotes samples from which we were able to isolate infectious virions after passaging fecal material in caco2 cells.

Influenza viral RNA was detected in stool samples of influenza-infected individuals; however, the impact of virus shedding on gut microbiota has not been investigated [31,32]. Thus, we compared microbiota diversity and composition between individuals who shed the virus in their stool (shedders) and those who did not (non-shedders). Here, we were able to detect the influenza virus in fecal samples of 14 patients, of which 11 were H1N1-positive, and three were H3N2-positive (Table 1). As expected, we were able to detect significant differences in alpha diversity (*p =* 0.023) but not in beta diversity of gut microbiota between shedders and non-shedders of H1N1- and H3N2-infected patients (Figure 5A). In general, shedders were exhibiting much less diversity compared to non-shedders (Figure 5A). To our surprise, though, only *Bacteroides fragilis* species of *Bacteroidetes* were found to be significantly less abundant among the non-shedders (*p =* 0.037) in both groups (Figure 5B). In general, the relative abundance of the top 20 most abundant genera in shedders and non-shedders of both groups was relatively similar (Figure 5C). In all, *Prevotella* was the most abundant genus identified (Figure 6).

### 2.4. Association between Microbiota Composition and Virus Replication in Intestine

Recently, there has been increasing evidence of the ability of seasonal and pandemic influenza viruses to replicate in human intestines [36]. To further investigate the effect of the influenza virus shedding on gut microbiota, all fecal samples positive for influenza (n = 14) were subjected to virus isolation in caco2 cells. Following three passages, we were able to see the cytopathic effect in cell cultures of six samples. Of these, five samples belong to H1N1-infected patients, and one is from an H3N2-infected patient (Figure 6). Alpha diversity analysis of these samples revealed higher diversity (*p* = 0.0426; mean = 3.33, SD = 0.51) compared to shredders (mean = 3.54, SD = 0.69) (Supplementary Figure S1A). This higher diversity was mainly driven by the increase in the relative abundance of three genera: *Blautia* (*p* = 0.02) and an unclassified *Veillonellaceae* family (*p* = 0.04), both of which belong to *Firmicutes*; *Bilophila* genus (*p* = 0.0023) that belongs to *Proteobacteria* (Supplementary Figure S1B).

### 2.5. Association between Microbiota Composition and Severity of Respiratory Infection

Recent studies found that changes in gut microbiota could be related to increased risk of bronchitis and the development of severe respiratory illness [37,38]. Accordingly, we investigated the link between intestinal microbiota and the severity of influenza infection. Lower respiratory tract symptoms and manifestations such as pneumonia and bilateral pulmonary air space disease were reported in eight patients. Four of these were influenza B-positive patients, while the other four were H1N1-positive patients (Table 1). Notably, there were no significant differences in alpha diversity of intestinal microbiota of patients suffering from lower respiratory tract infection (LRTI) and patients with typical flu symptoms (Supplementary Figure S2). On the other hand, the comparison of intestinal bacterial composition revealed that one unclassified family of bacteroidales order is represented at a significantly higher abundance among LRTI patients (*p* = 0.035) (Supplementary Figure S2).

## 3. Discussion

Recently, several reports have investigated the complex relationship of the gut–lung axis, particularly during viral respiratory infection [35]. Intestinal and respiratory tracts are thought to share a “common mucosal response”; that is, infection in either mucosal site can shape immune function at distant mucosal sites (intestine) [39]. The current study helps in understanding the adverse effects of influenza infection on the diversity and composition of gut microbiota. To our best knowledge, no other study has investigated and compared the impact of influenza types (A and B) and influenza A subtypes (H1N1 and H3N2) on intestinal microbiota. The overall bacterial diversity has increased slightly in influenza patients compared to healthy controls. Similar findings were reported in infants suffering from non-viral bronchiolitis [37,38,40]. In contrast to our results, studies investigating intestinal bacterial diversity in H7N9-infected patients and influenza A-infected mice reported an overall depletion of intestinal bacterial content [38,40]. The lower diversity in the latter studies could be partially explained by the ability of avian influenza viruses such as H7N9 and seasonal influenza viruses to replicate in human and mice intestines, respectively. Virus replication in these highly colonized surfaces should be expected to dramatically affect the richness of its bacterial content. The ability of seasonal H1N1 and H3N2 viruses to replicate in the human intestine, however, is still being investigated [31,41]. Disruption in gut microbiota could also result from the indirect effect of the immune response in the lungs. Studies in mice have linked changes in intestinal microbiota to immune response driven by type I and type II interferons secreted during influenza infection [42,43]. Our data, in addition to others, implicate those local intestinal diseases will alter gut microbiota diversity [44,45,46,47].

Defining the microbiome profile of healthy individuals is generally difficult due to inter-individual variations; however, typical intestinal microbiota belongs mainly to Firmicutes and Bacteroidetes phyla [48]. Altering the balance of these two phyla has been previously linked to various diseases [49]. Here, we noticed an overall increase in the abundance of Bacteroidetes with a corresponding decrease in the Firmicutes in influenza-infected patients. Interestingly, though, bacteria that caused these differences among groups varied at the family and genus levels between influenza A- and influenza B-infected individuals emphasizing that the observed profiles are influenza type dependent. In line with our findings, the Bacteroides-dominant profile was reported in mice infected with RSV and influenza A virus as well as in infants with non-viral bronchiolitis [37,42,50]. Therefore, further investigation is required to identify the association between Bacteroidetes and respiratory infections. An alternative explanation for the shift in Bacteroidetes and Firmicutes ratio is the infection-related gastrointestinal symptoms. Gastrointestinal symptoms have been reported in influenza-infected patients, particularly during the 2009 H1N1 pandemic [31]. Infection-induced anorexia was reported in patients following the influenza virus and RSV infection [51,52]. Such symptoms are most likely to be associated with changes in diet, which is the main contributor to microbiota composition [50,53]. In humans, lower food intake has been linked to an increase in Bacteroidetes abundance over Firmicutes [54,55], similar to what was observed in this study. In addition to the overall increase in Bacteroidetes, differential testing revealed more than ten differentially abundant OTUs infected individuals compared to healthy controls. Further analysis showed that most of these differences in gut microbiota composition were influenza type dependent. Notably, ten OTUs were found to be differentially abundant amongst influenza A- and influenza B-infected patients.

Influenza virus shedding in the stool of infected individuals has been reported in several studies [22,32,41]. In this study, the virus was detected in stools of 50% and 55% and H3N2- and H1N1-infected individuals, respectively. More importantly, we were able to isolate infectious viruses from samples of six patients. Remarkably, bacterial diversity in shedders was significantly lower compared to non-shedders regardless of the influenza A subtype. The presence of virus antigens or, more importantly, infectious virions in the intestine of shedders may trigger the immune response and subsequently resulting in less bacterial diversity in these patients. Surprisingly, though, differential abundance analysis of the two groups revealed only one differentially abundant OTU, Bacteroides fragilis, which belongs to Bacteroidetes. A high abundance of Bacteroides fragilis was also observed in fecal samples of infants—less than one month of age—infected with influenza [56]. Twenty-six percent of B. fragilis strains can synthesize a polysaccharide (PSA) that has an immunomodulatory effect and has been shown to repress colitis in an animal model [57,58]. In contrast, some B. fragilis strains produce an enterotoxin that can induce colitis in animal models as well as in humans [59]. Therefore, determining geno- and phenotype of B. fragilis strains from shedder is necessary to study the correlation between the increase in B. fragilis abundance and the shedding of the virus.

Further analysis of fecal samples of shedders revealed a significantly higher abundance of three genera: Blautia, Bilophila, and an unclassified genus of Veillonellaceae family. Blautia genus belongs to the Lachnospiraceae family, which is one of the most predominant families of mucosa-associated bacteria [60]. These mucus-associated bacteria express high levels of mucin utilization genes and can metabolize mucin glycans and use them to produce butyrate [61]. Butyrate is utilized as an energy source by colonic cells and to modulate immune response [62]. Therefore, the increased abundance of Blautia in fecal samples of these patients may indicate their role in restoring immune homeostasis in the presence of replicating the virus. A higher abundance of intestinal Blautia has usually considered an indication of better health compared to people with chronic illnesses such as cancer and diabetes [63]. The survival of the virus in the intestinal environment could be due to the swallowing of excess mucus from respiratory secretions. The increased abundance of the Veillonellaceae family, which is usually found in the mouth, could further support this assumption [64]. Increased abundance of Bilophila, on the other hand, has been reported in patients suffering from colitis due to their ability to induce pro-inflammatory response [65].

Severe respiratory symptoms were reported in 20% of patients enrolled in this study. Differential analysis of the intestinal bacterial content of these patients revealed a higher relative abundance of specific genera. The higher abundance of Bacteroidetes in influenza-positive patients in general and patients with severe respiratory symptoms, in particular, was driven by the increase in Bacteroidales order. As mentioned earlier, the Bacteroidetes-dominant profile was reported in infants suffering from bronchiolitis [37]. Studies have reported the role of Bacteroidales in controlling host immune response during infections [57]. However, they also can confer harmful effects on the host depending on their genetic characteristics [66]. Therefore, further characterization of identified Bacteroidales species is essential to explain their higher abundance in patients with severe symptoms. Patients with severe symptoms also exhibited a higher abundance of Firmicutes represented by the Enterococcus genus and an unclassified genus of the Veillonellaceae family. Enterococcus is normally found in the fecal contents of healthy individuals; however, when densely colonized in the intestine, it can cause bacteremia and endocarditis [67]. Although none of our patients suffered from any of these serious complications, the increased abundance of Enterococcus in patients with severe respiratory symptoms is a warning sign that should be considered.

In conclusion, increased Bacteroides–Firmicutes ratio was the dominant profile seen among infected individuals. However, whether Bacteroidetes-dominant profiles resulted from respiratory influenza infection, or their increased abundance rendered the host susceptible to respiratory infections, requires further investigations. Additionally, we cannot exclude other factors that might affect intestinal microbial communities, such as diet and genetic variabilities. The primary limitation of this study is the sample size of sub-groups (influenza A vs. influenza B and H1N1 vs. H3N2); therefore, a follow-up study with an increased sample size is underway.

## 4. Materials and Methods

### 4.1. Study Population and Sample Collection

This study was conducted to examine the impact of respiratory influenza infection on intestinal microbiota in male adults (median age = 31) with confirmed influenza infection (*n* = 38) compared to healthy individuals (*n* = 11) (Table 1). Participants, both patients and controls, are mainly of Asian origin (31% Indians, 24% Srilankan, 21% Filipinos, 15% Pakistanis). Paired nasal and fecal samples were collected during the influenza season from October 2018 through April 2019. Controls were healthy individuals with no chronic medical illnesses, aged between 18 and 60, and had not had any influenza vaccine in the last four years. Exclusion criteria were as follows: (i) patients with chronic heart, liver, or digestive tract diseases; (ii) diabetics; (iii) patients who received antibiotics in the last 2 years. Nasal and fecal samples were collected simultaneously during the hospitalization of infected patients and at the home of healthy individuals, following the standardized protocols of each sample type [68]. Briefly, nasal swabs were collected in viral transport media, while fecal samples were collected in sterile containers. Approximately 1 g of fecal sample was homogenized in 10% PBS, and aliqoted into two tubes. For bacterial DNA extraction, 10% glycerol was added to one of the aliquoutes and stored at −80 °C. For virus detection, resuspended fecal samples were centrifuged twice for 20 min at 3000 g, and the supernatant was collected and stored at −80 °C until used for viral RNA extraction.

### 4.2. Detection of Influenza Virus in Nasal and Fecal Samples

Viral RNA was purified from nasal swab specimens and stool specimens using the QIAamp viral RNA mini kit (Qiagen, Hilden, Germany) as instructed by the manufacturer. Identification of influenza type and influenza A subtype was performed using quantitative real-time PCRs as previously described [69,70].

### 4.3. Isolation of Influenza Virus from Fecal Samples

Influenza-positive fecal samples were selected for cell culture isolation in human epithelial colon carcinoma cell line (caco-2) cells. Fecal suspensions were filtered twice using 0.22 µL filters and added to 90% confluent caco2 cells cultured in infection medium (IM) containing 0.3% bovine serum albumin (BSA) and 1 µg/µL TPCK-trypsin). Cells were incubated at 37 °C for 7 days and checked daily for the presence of the cytopathic effect. At the end of this period or when the cytopathic effect appeared, supernatants were collected and tested for the presence of influenza A using real-time PCR. Negative samples underwent a maximum of three passages, and the results were recorded.

### 4.4. Bacterial DNA Extraction, 16S rRNA PCR and Sequencing

Bacterial DNA was extracted from fecal samples using the QIAamp UCP pathogen mini kit according to manufacturer protocol (Qiagen, Hilden, Germany). For purification of microbial DNA from stool samples, pre-lysis of bacterial suspension with the Pathogen Lysis Tubes was performed first before proceeding to column purification. Amplification of the 16S ribosomal RNA V4 region (~600 bp) was performed using barcoded primers: 515F 5′-GTGCCAGCMGCCGCGGTAA-3′, and 806R 5′-GGACTACHVGGGTWTCTAAT-3′, as previously described [71]. Purified PCR products were then subjected to pooled sequencing in the MiSeq platform using a 300-bp paired-end protocol. Sequencing reads were then trimmed, merged, demultiplexed, and quality filtered to remove noisy reads. Filtered, high-quality sequencing reads were classified into operational taxonomic units (OTUs) at 97% similarity threshold using the recommended procedure (https://www.mothur.org/wiki/MiSeq_SOP; accessed on 2 October 2019). Reads were mapped to the Greengenes v. 13_8 database (http://greengenes.secondgenome.com (accessed on 2 October 2019)) containing only the 16S V4 region to determine taxonomies and the consensus taxonomy for each OTUs was retrieved. A rarefied OTU table was then generated before proceeding to downstream diversity analyses. As a quality control, two template-free controls were extracted, amplified, and sequenced the same way as the samples. OTUs were discarded if their mean abundance in controls exceeds 25% of their mean abundance in samples.

### 4.5. Statistical Analysis

The richness and evenness (alpha diversity) of OTUs obtained from each sample was measured using raw OTU abundance tables and represented by the Shannon index. The significant differences in alpha diversity were tested with the two-way repeated-measures analysis of variance (ANOVA). We also estimated beta diversity., The distance matrix was calculated using the Bray–Curtis dissimilarity index to measure beta diversity, which was then visualized using Principal Coordinate Analysis (PCoA) ordination. Significant differences in gut microbiota community structure between the groups were calculated using permutational multivariate analyses of variance (PERMANOVA) on the Bray–Curtis distance matrix [72]. Differential abundance testing of OTUs was calculated using the DESeq2 package available by R (v.1.19.1) to identify differentially abundant taxa among group variables [73]. Significance (adjusted *p*-value) was determined as *p* < 0.05. An analytical diagram summarizing the tools and statistical tests used to generate the data and figures of this study is displayed in Supplementary Figure S3.

## Figures and Tables

**Table 1:** Characteristics and outcomes of all participants enrolled in this study.

	Influenza Positive					Healthy Controls
	A				B	
	H1N1		H3N2			
	Shedders	Non-Shedders	Shedders	Non-Shedders		
Number of individuals	11	9	3	3	12	11
Median age (IQR)	31.5 (15)	35 (7)	31	50	29.5 (10)	34 (8)
Fever (38–39 °C)	9	7	3	2	11	-
Lower respiratory tract infection	1	3	0	-	4	-

## Data Availability

The data presented in this study are openly available in NCBI (accession number PRJNA628513).

## Supplementary Materials

The following are available online at https://www.mdpi.com/article/10.3390/pathogens10060761/s1, Table S1: Differential abundance testing identified 15 OTUs that were differentially abundant amongst influenza positive patients and controls; Table S2: Differential abundance testing identified 10 OTUs that were differentially abundant amongst influenza A and influenza B infected patients; Figure S1: Analytical flowchart presenting a short guide to analysis and statistical tools used in this microbiome study; Figure S2: Statistical analysis of alpha diversity (A) and mean relative abundance (B) of OTUs identified in fecal samples of shedders and fecal samples from which infectious virion was isolated; Figure S3: Statistical analysis of (A) alpha diversity and (B) mean relative abundance of OTUs identified in patients suffering from severe respiratory infection and those with typical flu symptoms.
