# Supplementary material for: Profiling of Intestinal Microbiota in Patients Infected with Respiratory Influenza A and B Viruses
Source: Pathogens. 2021 Jun 17;10(6):761. doi: 10.3390/pathogens10060761 (PMC8233933; doi:10.3390/pathogens10060761)
Supplement: Supplementary file 1 [file pathogens-10-00761-s001.zip › pathogens-1242518-supplementary.pdf]

Supplementary Materials

Table S1. Differential abundance testing identified 15 OTUs that were differentially abundant amongst influenza positive patients and controls.

Bacterial taxa	Phylum	p-value	P-adj*	Higher in Infected	Higher in Controls
s__ *Prevotella copri*	*Bacteroidetes*	0.000	0.000	yes	
s__ *Faecalibacterium prausnitzii*	*Firmicutes*	0.000	0.000		yes
g__ *Dialister* unclassified	*Firmicutes*	0.000	0.002	yes	
f__ *Lachnospiraceae* unclassified	*Firmicutes*	0.000	0.005		yes
f__ *Barnesiellaceae* unclassified	*Bacteroidetes*	0.000	0.005		yes
f__ *Veillonellaceae* unclassified	*Firmicutes*	0.000	0.016	yes	
f__ *Enterobacteriaceae* unclassified	*Proteobacteria*	0.000	0.016		yes
s__ *Escherichia coli*	*Proteobacteria*	0.001	0.017		yes
s__ *Faecalibacterium prausnitzii*	*Firmicutes*	0.001	0.017		yes
f__ *Lachnospiraceae* unclassified	*Firmicutes*	0.001	0.023		yes
f__ *Victivallaceae* unclassified	*Lentisphaerae*	0.001	0.026		yes
f__ *Lachnospiraceae* unclassified	*Firmicutes*	0.001	0.026		yes
g__ *Dialister* unclassified	*Firmicutes*	0.001	0.026	yes	
g__ *Dialister* unclassified	*Firmicutes*	0.001	0.026	yes	
f__ *Lachnospiraceae* unclassified	*Firmicutes*	0.001	0.028	yes	

* P-adj: Adjusted p-value. Only significant adjusted p-values of less than 0.033 are presented in this table. S for species, g for genus and f for family.

Table S2. Differential abundance testing identified 10 OTUs that were differentially abundant amongst influenza A and influenza B infected patients. .

Bacterial taxa	Phylum	p-value	P-adj*	Higher in Influenza A	Higher in influenza B
f__*Lachnospiraceae* unclassified	*Firmicutes*	0.000	0.002		yes
g__*Sutterella* unclassified	*Proteobacteria*	0.000	0.002	yes	
f__*Erysipelotrichaceae* unclassi-fied	*Firmicutes*	0.000	0.004		yes
g__*Roseburia* unclassified	*Firmicutes*	0.000	0.005		yes
f__*Lachnospiraceae* unclassified	*Firmicutes*	0.000	0.013		yes
g__*Megasphaera*_unclassified	*Firmicutes*	0.000	0.013	yes	
f__*Veillonellaceae*unclassified	*Firmicutes*	0.000	0.013	yes	
s__*Roseburia_faecis*	*Firmicutes*	0.001	0.015		yes
f__S24-7/ *Muribaculaceae* un-classified	*Bacteroidetes*	0.001	0.015		yes
f__*Lachnospiraceae*_unclassi-fied	*Firmicutes*	0.001	0.015		yes

* P-adj: Adjusted p-value. Only significant adjusted p-values of less than 0.033 are presented in this table. S for species, g for genus and f for family. .

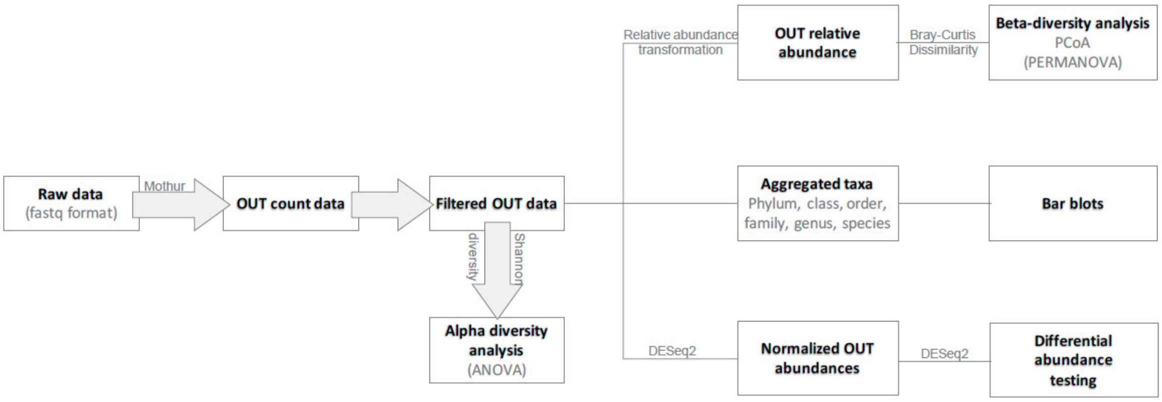

Figure S1. Analytical flowchart presenting a short guide to analysis and statistical tools used in this microbiome study.

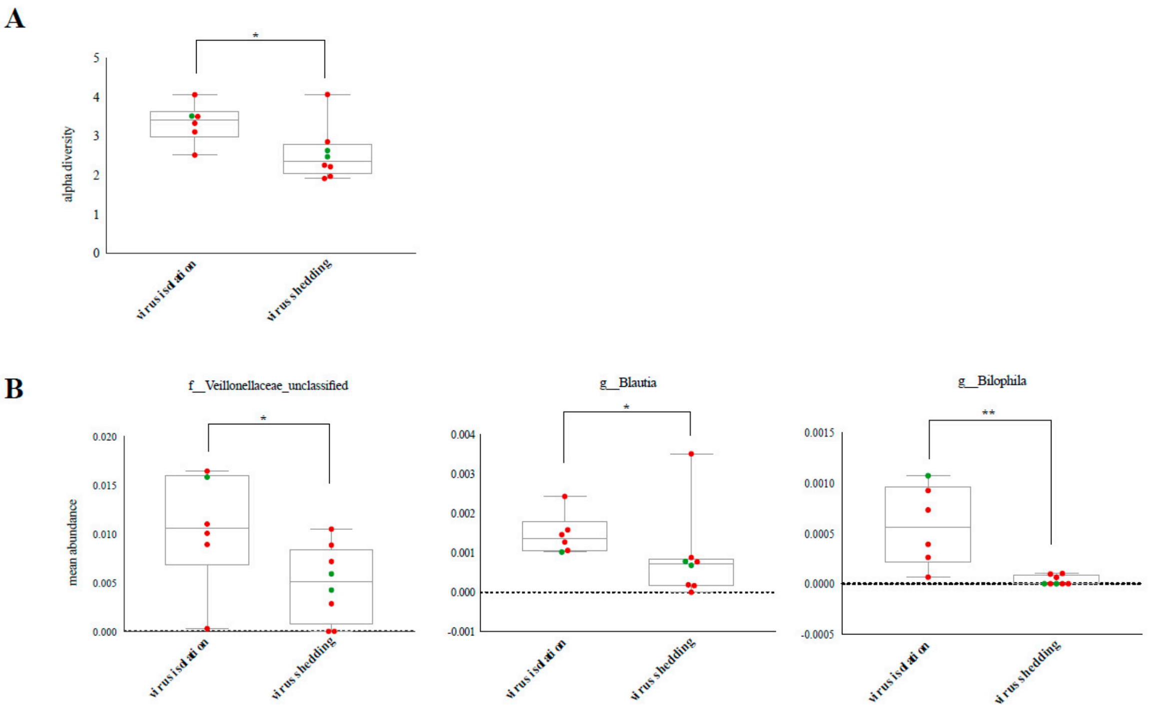

Figure S2. Statistical analysis of alpha diversity (A) and mean relative abundance (B) of OTUs identified in fecal samples of shedders and fecal samples from which infectious virion was isolated. (A) Comparison of alpha diversity between the two groups. Dots represents samples and are color-coded as follows: red for H1N1 and green for H3N2. (B) Comparison of mean relative abundance of all genera identified in samples of both groups. P values less than 0.05 are indicated with one asterisk, and P values less than 0.01 are summarized with two asterisks. Only genera that showed significance ($p < 0.05$) are presented here.

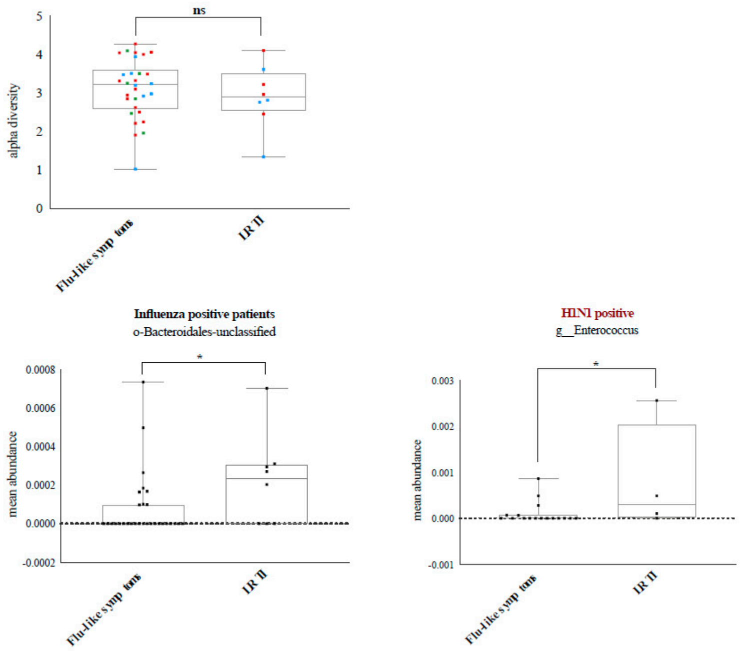

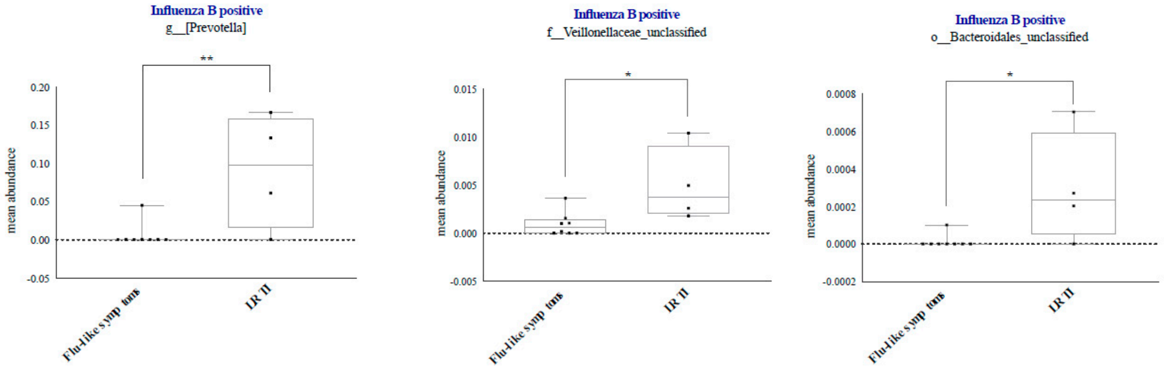

Figure 3. Statistical analysis of (A) alpha diversity and (B) mean relative abundance of OTUs identified in patients suffering from severe respiratory infection and those with typical flu symptoms. (A) Comparison of alpha diversity of patients suffering from lower respiratory tract infection (LRTI) and patients with typical flu symptoms (fever, cough, sore throat and body pain). Dots represents samples and are color-coded as follows: red for H1N1, blue for influenza B and green for H3N2. (B) Comparison of mean abundances of each of identified genera between patients with LRTI and patients with typical flu symptoms. P values less than 0.05 are indicated with one asterisk, and P values less than 0.01 are summarized with two asterisks. Only genera that showed significance ($p < 0.05$) are presented here.
